# Update on Acute Bone and Joint Infections in Paediatrics: A Narrative Review on the Most Recent Evidence-Based Recommendations and Appropriate Antinfective Therapy

**DOI:** 10.3390/antibiotics9080486

**Published:** 2020-08-06

**Authors:** Giovanni Autore, Luca Bernardi, Susanna Esposito

**Affiliations:** Pediatric Clinic, Pietro Barilla Children’s Hospital, Department of Medicine and Surgery, University of Parma, 43126 Parma, Italy; giovanniautore@gmail.com (G.A.); bernardi.luca91@gmail.com (L.B.)

**Keywords:** antibiotic, bone infection, joint infection, osteomyelitis, pediatric infectious disease, septic arthritis

## Abstract

Acute bone and joint infections (BJIs) in children may clinically occur as osteomyelitis (OM) or septic arthritis (SA). In clinical practice, one-third of cases present a combination of both conditions. BJIs are usually caused by the haematogenous dissemination of septic emboli carried to the terminal blood vessels of bone and joints from distant infectious processes during transient bacteraemia. Early diagnosis is the cornerstone for the successful management of BJI, but it is still a challenge for paediatricians, particularly due to its nonspecific clinical presentation and to the poor specificity of the laboratory and imaging first-line tests that are available in emergency departments. Moreover, microbiological diagnosis is often difficult to achieve with common blood cultures, and further investigations require invasive procedures. The aim of this narrative review is to provide the most recent evidence-based recommendations on appropriate antinfective therapy in BJI in children. We conducted a review of recent literature by examining the MEDLINE (Medical Literature Analysis and Retrieval System Online) database using the search engines PubMed and Google Scholar. The keywords used were “osteomyelitis”, OR “bone infection”, OR “septic arthritis”, AND “p(a)ediatric” OR “children”. When BJI diagnosis is clinically suspected or radiologically confirmed, empiric antibiotic therapy should be started as soon as possible. The choice of empiric antimicrobial therapy is based on the most likely causative pathogens according to patient age, immunisation status, underlying disease, and other clinical and epidemiological considerations, including the local prevalence of virulent pathogens, antibiotic bioavailability and bone penetration. Empiric antibiotic treatment consists of a short intravenous cycle based on anti-staphylococcal penicillin or a cephalosporin in children aged over 3 months with the addition of gentamicin in infants aged under 3 months. An oral regimen may be an option depending on the bioavailability of antibiotic chosen and clinical and laboratory data. Strict clinical and laboratory follow-up should be scheduled for the following 3–5 weeks. Further studies on the optimal therapeutic approach are needed in order to understand the best first-line regimen, the utility of biomarkers for the definition of therapy duration and treatment of complications.

## 1. Introduction

Acute bone and joint infections (BJIs) in children may clinically occur as osteomyelitis (OM) or septic arthritis (SA). BJIs generally present clinically within 2 weeks of disease onset [1]. In clinical practice, one-third of cases present a combination of both conditions, and this combination may occur in as many as 75% of cases in newborns [2]. BJIs are usually caused by the haematogenous dissemination of septic emboli carried to the terminal blood vessels of bone and joints from distant infectious processes during transient bacteraemia. Less common infection routes are direct inoculation due to open fractures or invasive procedures and extension from contiguous infections, such as cellulitis and sinusitis. BJI can be classified as acute, subacute and chronic according to its duration: <2 weeks, <3 months and >3 months from onset, respectively. Chronic infections are relatively rare conditions in paediatric patients, could be caused by the establishment of biofilm, and different surgical approaches must be considered [3].

The mean annual incidence of BJI in high-income countries is approximately 8 per 100,000 children [4,5]. Despite the high variability between different reports, an increasing trend has been observed over the last few decades, probably due to increased diagnostic effectiveness. Gafur et al. observed that the annualised per capita incidence of OM increased 2.8-fold in the same paediatric hospital within two decades [4]. Children aged ≤ 5 years showed a higher prevalence, accounting for half of all cases [6]. Although uncommon, BJI in children should not be underestimated because local and systemic complications may result in life-threatening conditions and severe disabilities. If not promptly diagnosed and treated, the infection may extend to soft tissues, causing pyomyositis (especially in young infants) and sepsis [7]. Local progression may result in subperiosteal or intraosseous abscesses, pathological fractures, and abnormal bone growth due to the involvement of the epiphysis [8,9,10]. Venous thrombosis and septic embolism may also occur, more commonly in children aged >8 years [11,12].

Early diagnosis is the cornerstone for the successful management of BJI, but it is still a challenge for paediatricians, particularly due to its nonspecific clinical presentation and to the poor specificity of the laboratory and imaging first-line tests that are available in emergency departments. Moreover, microbiological diagnosis is often difficult to achieve with common blood cultures, and further investigations require invasive procedures. The indications and effectiveness of available diagnostic tools are still debated. In addition, the resistance pattern of aetiological agents and poor bone penetration of antibiotics represent a challenge for an appropriate therapeutic approach. Therefore, the aim of this narrative review is to provide the most recent evidence-based recommendations on appropriate antinfective therapy in BJI in children. 

## 2. Aetiology and Pathogenesis

BJI commonly occurs in primarily healthy children without clear predisposing conditions. However, a higher prevalence is described in patients affected by immunodeficiencies and haemoglobinopathies, particularly sickle cell disease (SCD) and chronic granulomatous disease (CGD) [13,14]. Previous experimental studies on animal models have suggested that minor trauma may increase the susceptibility of bone and joint tissues to bacterial seeding [15]. More recent clinical studies question this association because children affected by BJIs show the same rate of previous minor trauma observed in the general paediatric population [16].

More than 80% of the cases of OM occur in the metaphysis of long tubular bones, and the most common localisations are the femur and tibia, followed by the pelvis and calcaneus among nontubular bones [17,18]. A nationwide survey conducted in the USA revealed that osteomyelitis in the pelvis, upper arm, hand and forearm was associated with a higher risk of septic arthritis and bacteraemia or septicaemia [19]. The knee and hip are the most commonly involved joints, accounting for more than half of SA cases [18]. In a large multicentre study recently conducted in Spain, the involvement of the hip in children affected by combined osteomyelitis and septic arthritis was the main negative prognostic factor associated with a higher risk of complications and sequelae [20].

The choice of proper empiric treatment is the main issue in the management of paediatric BJI. First-line antibiotics should cover the most likely aetiologies according to the patient’s age and local prevalence of community-acquired and nosocomial pathogens. In addition, they should have an appropriate bone penetration. According to the largest studies in recent literature, methicillin-sensitive *Staphylococcus aureus* (MSSA) may still be considered the most common pathogen in Europe, with a prevalence ranging from 30% to 63% of confirmed cases [5]. However, the emerging role of *Kingella kingae* has been confirmed by several studies reporting its isolation in up to 53% of all cases [5]. In addition, *Streptococcus pneumoniae* and Group A β-haemolytic *Streptococcus pyogenes* (GABHS) maintain a relevant role in patients aged ≥ 6 months. On the other hand, the prevalence of *Haemophilus influenzae* type b has drastically decreased in the post-vaccination era, accounting for less than 1% of cases [21].

The aetiology, however, largely depends on the patient’s age (Table 1). A recent multicentre study conducted in France on 71 patients aged under 3 months reported that *Streptococcus agalactiae* is the main community-acquired pathogen accounting for 45% of these cases, followed by *S. aureus* (22% of all cases) which was the most frequent microorganism in infants aged over 2 months, and *Escherichia coli* (18%) [22]. These findings were confirmed by Juchler et al. in infants aged 0–6 months, reporting *S. aureus*, *S. agalactiae*, and *E. coli* as the most frequently isolated pathogens, accounting for 31%, 15%, and 8% of these cases, respectively [23]. In this age group, both studies observed that *Klebsiella pneumoniae* and *Candida albicans* are particularly frequent nosocomial pathogens, and they each account for 7% of cases [22]. Among children aged between 6 months and 5 years, *K. kingae* is the most frequent cause of osteoarticular infections, accounting for half of these cases [23]. Less frequent pathogens in this age group are *S. pneumoniae* and GABHS, which were isolated in 29% and 7% of cases, respectively [23]. In the post-vaccination era, *S. pneumoniae* still represents a causative agent of septic arthritis, accounting for over 4% of all cases [4,17]. The detection of *K. kingae* requires specific PCR assays. *K. kingae* seems to be very specific for the age group under 5 years. In fact, a large review of 566 osteoarticular infections caused by this pathogen reported that 80% of these cases occurred in children aged under 4 years [24]. Moreover, Ferroni et al. observed a higher prevalence of *K. kingae* among children affected by SA than OM [25]. A prospective case-control study revealed that oropharyngeal carriage of *K. kingae* is strongly associated with haematogenous BJI in children aged under 4 years. Using a specific polymerase chain reaction (PCR) assay, the authors identified the microorganism in the oropharynx of 71% of previously confirmed cases of osteoarticular infections and only in 6% of age-matched healthy controls, reporting an odds ratio of approximately 38.3 (95% CI, 18.5–79.1) [26].

BJIs in children aged over 5 years are most frequently caused by *S. aureus*, which accounts for up to 61% of these cases [23]. *K. kingae* is isolated in less than 13% of cases in this age group, followed by GABHS [20,23]. Once considered mainly nosocomial, the prevalence of particularly virulent strains of community-acquired *S. aureus* (CA-SA) is increasing, causing severe forms of BJI. Approximately 70–90% of confirmed cases caused by CA-SA involve methicillin-sensitive strains (MSSA), but there has been an increase in cases of BJI from community-acquired methicillin-resistant *S. aureus* (CA-MRSA) [27,28]. Studying the regional prevalence of CA-MRSA is mandatory because, according to the recent guidelines published by the European Society for Paediatric Infectious Diseases (ESPID), a local prevalence over 10% should induce clinicians to choose a different empiric treatment from conventional first-line drugs. Another virulence factor causing severe forms of staphylococcal infections with issues of management is Panton–Valentine leukocidin (PVL). PVL is a bicomponent, pore-forming toxin produced by some strains of *S. aureus* (PVL-SA) that kills leukocytes. A multicentre European study conducted in 7 countries reported that PVL-SA reached an incidence up to 18%, and PVL was produced more frequently by MSSA strains in Europe, whereas it was more common among MRSA strains in the United States [29]. In the same study, MRSA isolates represented 6% of all cases caused by CA-SA [29]. Nationwide studies conducted in Spain and the UK observed rates of CA-MRSA ranging from 2.5% to 3% of all community-acquired staphylococcal infections, while PVL-SA has been isolated in 10% of these cases in Italy [20,30]. CA-MRSA seems to be less prevalent in European populations than in the USA, where its prevalence reached 30% in some regions within the last two decades [31].

## 3. Clinical Presentation

Onset symptoms of BJI are usually nonspecific in children. Children with osteomyelitis usually present acutely with fever and constitutional symptoms, such as irritability and decreased activity. Once the infection progresses, focal symptoms and signs of bone inflammation may occur. According to a large systematic review conducted over a population of 12,000 children with BJI, the most common onset features included localised pain (81%); focal warmth, swelling and point tenderness (70%), fever (62%) and limitation of function (50%) [17]. Clinical suspicion may be difficult in newborns and toddlers because they often lack focal findings and may continue to feed well. Although osteomyelitis is rare in this age group, it should be considered for the differential diagnosis in infants with skin infections, urinary tract anomalies, prematurity, and neonatal sepsis. Mediamolle et al. reported that 94% of infants aged under 3 months showed pain, and 87% of them had functional limitations, but only 52% were febrile [22]. BJI should also be suspected in older children presenting with fever without a source, bacteraemia, and abnormal radiological findings in the evaluation of trauma. A recent history of infections is uncommon, and this makes it difficult to identify the initial source of haematogenous dissemination. Ferroni et al. were able to tentatively identify the portal of entry in only 55% of cases, of which 55% were upper respiratory tract infections; 15%, skin trauma; 11%, gastro-enteritis; 8%, varicella; and 2%, congenital infections [25].

Special populations of children at higher risk of BJI may present with atypical features. Patients with haemoglobinopathy (i.e., sickle cell disease) may present multifocal infections that are difficult to distinguish from vaso-occlusive crisis [13]. Children with chronic granulomatous disease may have BJIs caused by uncommon bacterial or fungal pathogens that usually remain asymptomatic even in advanced stages [14].

When OM is suspected, the differential diagnosis should include traumatic fracture, cellulitis or pyomyositis, rheumatic fever, thrombophlebitis, leukaemia, tumours, sickle cell infarction, tuberculosis, scurvy, and other bone inflammatory processes such as hypophosphatasia and chronic recurrent multifocal osteomyelitis (CRMO). Sepsis should also be ruled out in neonates. Moreover, some clinical signs of SA may also occur in cases of transient synovitis, viral arthritis, reactive arthritis, juvenile idiopathic arthritis, Henoch–Schoenlein purpura, and Perthes disease.

## 4. Diagnosis

### 4.1. Blood Examination

Initial blood tests for children with suspected BJI include the complete blood count (CBC), erythrocyte sedimentation rate (ESR), and level of C-reactive protein (CRP) [32]. Elevated ESR and CRP show a high sensitivity at disease onset but a low specificity. Dartnell et al. observed that CRP was elevated in 81% of patients at the time of presentation, with a peak on the second day from onset; instead, ESR peaked 3–5 days after onset [17]. The same study showed that the WBC count was elevated in only 36% of children at the time of diagnosis [17]. However, the CBC is helpful in the differential diagnosis of children with bone pain (e.g., leukaemia). A prospective study of 265 children with osteomyelitis and septic arthritis reported a mean CRP value of approximately 87 mg/L and a mean ESR value of approximately 51 mm/h at the time of presentation [33]. The combination of elevated CRP and ESR showed the best sensitivity to suspect BJI in children [33]. Several studies observed that CRP and ESR are higher and remain abnormal for a longer period in patients with MRSA infection [34]. In addition, MRSA is associated with greater elevations in CRP, ESR and WBC levels [18]. At this time, the role of procalcitonin is unclear, and its effectiveness compared to CRP is debated [32]. Blood culture should always be obtained at the same time as initial blood tests, as well as in afebrile patients, if the clinical suspicion of BJI is well-founded [32].

### 4.2. Imaging

The initial imaging study should be the radiograph of the suspected area in order to exclude other causes of pain [32]. However, radiographs are usually normal at the beginning of BJIs, and other advanced techniques are often required. Exceptions may be presented by newborns, who more commonly show abnormal radiographs at the onset [35].

The main X-ray features that suggest a BJI are periosteal reaction, periosteal elevation (suggesting a periosteal abscess), lytic lesions or sclerosis, and narrowing of the intervertebral disc space. At the onset, these alterations are often undetectable, and the timing of radiographic changes depends on the involved bones and the age of the patient. In long bones, cortical thickening and periosteal reaction/elevation are shown only 10 to 21 days after the onset of symptoms (7 to 10 days in newborns) [36]. Lytic sclerosis usually occurs only after more than a month. Due to the indolent course of the discitis that delays the onset of symptoms, X-ray signs of this category of BJI are often evident at the time of presentation [37].

Indications for additional imaging studies are confirmation of the diagnosis in clinically suspected BJI with normal radiographs, further evaluation of detected lesion and its extension (i.e., the involvement of epiphysis and adjacent soft tissues), surgical planning, and guidance for percutaneous procedures.

Magnetic resonance imaging (MRI) can be considered the gold standard imaging method for the diagnosis of BJI and to evaluate the involvement of surrounding soft tissues or joints [32]. The sensitivity and specificity of this technique range from 80% to 100% and from 50% to 100%, respectively [17]. The variability observed between the reported specificity rates may also depend on radiologist-specific experience. The use of intravenous gadolinium is not routinely required, but it is useful to detect intramedullary or muscular abscesses or necrosis, although it should be avoided in patients with renal insufficiency due to the risk of nephrogenic systemic fibrosis [38,39].

MRI can highlight multiple alterations that suggest the diagnosis of BJI. Areas of active inflammation show a decreased signal in T1-weighted images and an increased signal in T2-weighted images [40]. Fat-suppression sequences, including short-tau inversion recovery (STIR), decrease the signal from fat and are more sensitive for the detection of bone marrow oedema. In discitis, MRI easily detects the reduction of disc space, the increased T2-weighted signal in the adjacent vertebral endplates, and bone oedema in the vertebral body [38].

MRI is superior to other imaging methods, particularly to identify early infections affecting the bone marrow before the involvement of the cortical bone, to detect pelvic OM and discitis that are usually undetected by X-rays, to evaluate the involvement of the growth plates, joint structures, and soft tissues (e.g., pyomyositis, muscular abscesses) and to exclude deep venous thrombosis associated with BJI [41,42,43]. Furthermore, MRI is usually required in presurgical planning and surgical follow-up when drainage is indicated. MRI is also preferred because it prevents children from exposure to ionising radiation.

The main disadvantages of MRI are the longer scan time than CT and the need for sedation in young children. Furthermore, MRI is not always easily available, and it is more expensive. Because of its high sensitivity, the diagnosis of BJI is unlikely if the MRI is negative [44]. False-positive results can occur in patients with primary infections in adjacent soft tissues.

Regarding other imaging methods, computerised tomography (CT) scans are not generally recommended because they are less sensitive than MRI and expose children to ionising radiation. It should be considered in diagnosis only when MRI is not feasible [32]. However, CT scans may play a role when there is important bone destruction on radiographs to assess the extent of bone damage for a surgical approach [41]. It should also be useful in chronic OM when inflammation is too weak to be detected by MRI. In these cases, a CT scan can be performed without sedation and takes less time than MRI. BJI can be detected by the evidence of increased bone marrow density, new periosteal bone formation with periosteal purulence and irregular erosion of bone surfaces.

Bone scintigraphy is used to identify multifocal OM or when localised signs of bone involvement are too poor. It may be more accessible than MRI, and sedation is required less frequently. Technetium radionuclide scanning (^99m^Tc) has high sensitivity but lower specificity compared to MRI; furthermore, scintigraphy has proven to be scarcely sensitive (53%) for OM caused by MRSA [44,45]. ^99m^Tc scanning is triphasic, consisting of the flow phase (2 to 5 s after injection), blood pool phase (5 to 10 min after injection), and delayed phase (2 to 4 h after injection). BJI causes focal absorption in the third phase, and signal intensity is related to the level of osteoblastic activity. Localisation of a lesion near a growth plate can complicate the interpretation. Using ^99m^Tc-methylene diphosphonate (^99m^Tc-MDP), early evidence of infection can be detected 24 h after onset. Hsu et al. observed that specificity may increase with Gallium scanning and In-labelled leukocytes, although these techniques are more complex and add higher radiation exposure [46]. The disadvantages of scintigraphy are the lack of information on the size of pus collections that could be drained (i.e., in cases of intramedullary abscess), exposure to ionising radiation, and false-negative results that may occur if the blood flow to the periosteum is interrupted (i.e., in cases of subperiosteal abscess) [44].

Ultrasonography (US) is not useful for the diagnosis of BJI. With US, it is possible to identify the fluid collections in soft tissues associated with bone infections, and US can be a support for percutaneous diagnostic and therapeutic drainage [47]. Table 2 summarises indications and features of imaging methods in bone and joint infection in pediatric age.

### 4.3. Microbiological Diagnosis

With the increasing prevalence of antibiotic-resistant microorganisms and emerging pathogens, it is important to collect as many microbiological specimens as possible, and different microbiological tests are often required (Table 3). A microbiological diagnosis is achieved in barely more than half of all cases. In their systematic review, Dartnell et al. reported that microbiological diagnosis is achieved in approximately 50% of all cases of BJI [17]. In the context of a paediatric emergency department, Akinkugbe et al. reported microbiological isolation in only 38% of their cases [30]. On the other hand, a significantly higher success rate, approximately 70%, was reported in a population of patients aged under 3 months [22]. Calvo et al. also observed that the rate of microbiological diagnosis reached 61% in the combined form of OM and SA [20].

Blood culture has the lowest sensitivity, but it is also the most accessible technique. Reported rates of positive blood cultures are highly variable in the literature. McNeil et al. estimated a general sensitivity of approximately 46%, and other studies observed even lower rates for SA [20,48]. Juchler et al. observed that sensitivity may be increased by performing PCR assays on negative cultures; in this way, the authors reported an increase of +4.5% [23]. However, according to the ESPID guidelines, blood culture should always be analysed in cases where there is clinical suspicion (including afebrile patients); the culture should be performed at the same time as the initial laboratory evaluations and should be repeated at fever peaks [32].

When joint involvement is clinically suspected, synovial fluid can be obtained for microbiological analysis. For easily accessible joints (e.g., knee), arthrocentesis may be performed under conscious sedation in the emergency room. Less accessible joints, such as the ankle and hip, need an interventional radiologist. The sensitivity of synovial fluid culture is higher than that observed for blood culture and can be higher than 50% [48]. Arthrocentesis should always be performed when the involvement of accessible joints is clinically evident, and it should also be considered in complicated or nonresponsive cases affecting proximal joints. In order to increase the diagnostic yield of joint aspirate, synovial fluid can be inoculated in blood culture vials.

Bone samples can be obtained with a minimally invasive percutaneous needle biopsy, especially when subperiosteal abscesses occur, or with surgical biopsy in the operating room. The reported sensitivity rates for these invasive techniques may reach 82%, but they expose patients to higher risks [48]. Bone biopsy should be performed in complicated cases with negative blood culture that do not respond to empiric treatment, but it may also be considered when a bone abscess is easily accessible [32]. In fact, for most uncomplicated BJIs, invasive biopsy does not affect the clinical outcomes [49,50]. Nevertheless, surgical biopsy should be performed in every patient who undergoes surgical treatment. In their large prospective study, Ferroni et al. performed arthrocentesis for every SA and bone biopsy only when a subperiosteal abscess occurred; in this way, the authors reported high rates of microbiological isolation, reaching 40% for synovial fluid and 87% for bone samples [25].

Nucleic acid amplification methods, such as conventional and real-time polymerase chain reaction (PCR), improved the detection of pathogens even after the administration of antibiotics. Synovial fluid PCR may remain diagnostic up to 6 days after the first antibiotic dose, and similar results have been observed for bone samples [51]. Specific PCR analysis may also be the only way to identify *K. kingae* and its isolation can be enhancement by inoculation of samples in blood culture vials [51,52].

## 5. Antinfective Treatment

Empiric antinfective treatment should be started as soon as BJI is clinically suspected. The choice of empiric antimicrobial therapy is based on the most likely causative pathogens according to patient age, immunisation status, underlying disease, and other clinical and epidemiological considerations, including the local prevalence of MRSA. In addition, antibiotic bioavailability and bone penetration should be considered [53]. Then, management is guided by the results of the antibiograms obtained from the microbiological investigations performed before starting antimicrobial therapy [36].

In neonates younger than two months, empirical treatment should be oxacillin or cefazolin and gentamicin to cover *S. agalactiae* and other gram-negative organisms that are common causes of BJI in this age group [22,54,55]. In children aged 3 months and over, anti-staphylococcal penicillin or a cephalosporin such as cefazolin or cefuroxime should be used to target MSSA, *S. pneumoniae*, GABHS and *K. kingae* [32]. Among the anti-staphylococcal penicillins, the use of flucloxacillin should be preferred because it is well tolerated and shows high bone penetration, even if it is difficult to use for the type of formulation.

In areas with a local prevalence of MRSA higher than 10%, the administration of empirical therapies active against these pathogens is indicated [32,49]. In these cases, the first-choice drugs are clindamycin, vancomycin or linezolid [32,56]. Peltola et al. suggested the empirical use of clindamycin in areas where the prevalence of MRSA is over 10%, and the clindamycin resistance rate is under 10% or vancomycin if the prevalence of MRSA is over 10% and the clindamycin resistance rate is over 10%, with linezolid as the second-line choice [49]. Dalbavancin, even as a single dose, appeared effective in children with BJI due to MRSA [57,58]. Compared to other available antibiotics that are active against MRSA, the advantages of dalbavancin include a lower potential for drug interactions and the possibility of fewer required doses due to a longer half-life [59]. Another second-line drug after the failure of previous antibiotics may be daptomycin [60,61]. In complicated severe cases, when the involvement of PVL SA is suspected, antibiotic therapy should aim to inhibit toxin production. In these cases, inhibitors of protein synthesis, such as clindamycin, linezolid, and rifampicin, are the first choice [62,63]. Among less common pathogens, *Salmonella* spp. is a frequent cause of BJI in developing countries and in patients with sickle cell anaemia, and it should be treated with a third-generation cephalosporin or fluoroquinolone [5]. Candida spp. is mainly isolated in spondylodiscitis and requires prolonged antifungal treatment and surgical debridement [5].

The total duration of antibiotic treatment is widely debated in the literature. Classically, BJIs are treated with long courses of intravenous therapy and prolonged hospitalisation, with OM usually treated for 3–6 weeks and SA for 2–4 weeks. Peltola et al. have shown that even 10 days of treatment is sufficient for SA [64]. Moreover, a recent paper from France has shown that 15 days of treatment is sufficient in most of the cases [65]. Another prospective French study on 70 cases reported no failures of treatment with an intravenous regimen prolonged up to 8 days [25]. A retrospective study was conducted in Spain on 607 children with a mean duration of intravenous therapy of 12.9 days and reported good outcomes [20]. A multicentre randomised trial was conducted in Finland on 252 children randomly assigned to two therapeutic groups. The treatment involved a common short cycle of 2–4 days of intravenous antibiotics for both groups, followed by oral therapy with clindamycin or a high-dose first-generation cephalosporin for 20 days in the first group or 30 days in the second group. The authors observed no significant differences between the two groups, suggesting the effectiveness of shorter treatment regimens [66]. However, a limitation of this study was the absence of cases due to MRSA or PVL-SA. When spondylodiscitis occurs, it is still recommended to carry out intravenous therapy for 1–3 weeks [66]. A similar observational study conducted in the United States showed excellent outcomes with an early transition to oral antibiotics within 4 days; the researchers reported no significant difference in the treatment failure rate compared to that with longer intravenous regimens [67]. Only in case of patients with infection due to PVL-SA prolonged antimicrobial treatment and multiple surgical procedures are recommended since these infections are often complicated with abscesses and venous thrombosis [64].

The timing for switching from an intravenous to an oral regimen is still debated. Clinical criteria are apyrexia, compliance with oral therapy, pain reduction, and both general and local clinical improvement. Clinical conditions should be in accordance with the reduction in inflammatory markers such as CRP, ESR, and WBC count. Different cut-offs have been proposed for the evaluation of laboratory markers. Some authors prefer to wait until the complete normalisation of CRP before switching the antibiotic regimen [68]. Faust et al. considered acceptable a CRP value under 20 mg/L or at least a decrease of 2/3 of its maximum peak [36]. The ESPID guidelines recommend switching to oral therapy only when the patient presents an improvement in clinical conditions without fever for at least 24 h and a decrease of 30–50% from the CRP maximum peak is observed. However, the guidelines suggested prolonging the intravenous regimen if drug-resistant or more virulent pathogens are isolated [35].

In most observational studies and randomised clinical trials, oral therapy consists of high-dose cephalosporin or clindamycin [59,69,70,71]. Trials conducted by Peltola et al. showed a failure rate under 1% at follow-up [64]. Trimethoprim/sulfamethoxazole (TMP/SMX) has been successfully used in oral treatment of BJI in children [72,73,74]. The duration of oral therapy in uncomplicated BJIs is frequently approximately 3–4 weeks with rigorous monitoring of inflammatory markers and drug tolerability [32]. In this way, therapy can be continued at home, allowing patient discharge and subsequent outpatient follow-up.

## 6. Conclusions

The clinical presentation of BJI in children may be nonspecific and paucisymptomatic, especially in newborns and immunocompromised patients. Indirect functional signs of OM or SA should be carefully evaluated. All children with negative or inconclusive initial radiographic examination should undergo further highly sensitive imaging studies, such as MRI or bone scintigraphy. MRI is the gold-standard imaging method. It should always be performed in a diagnostic dilemma when the initial radiograph is negative. Contrast enhancement is not routinely required.

Empiric antibiotic therapy should be started as soon as possible. The choice of empiric antimicrobial therapy is based on the most likely causative pathogens according to patient age, immunisation status, underlying disease, and other clinical and epidemiological considerations, including the local prevalence of virulent pathogens, antibiotic bioavailability and bone penetration.

Despite the high success rate reported with empirical therapies, aetiological diagnosis is highly recommended. Blood culture should be obtained in every patient (even if he/she is afebrile) at the initial evaluation and repeated at the fever peak. Synovial fluid samples should also be obtained in the case of SA if antibiotics have already been administered. Bioptic samples are not routinely required in uncomplicated BJI. Instead, minimally invasive percutaneous bone biopsy and surgical biopsy should be considered in complicated infections and when surgery is indicated.

Multidisciplinary management is necessary to achieve an early diagnosis. Paediatricians should consult an experienced radiologist (or an interventional radiologist if percutaneous procedures are indicated) and an orthopaedic surgeon. Microbiological laboratories should also be directly consulted if the involvement of pathogens that are difficult to isolate is suspected (e.g., *K. kingae*).

Empiric antibiotic treatment consists of a short intravenous cycle based on anti-staphylococcal penicillin or a cephalosporin in children aged over 3 months with the addition of gentamicin in infants aged under 3 months. An oral regimen may be an option depending on the bioavailability of the antibiotic chosen and clinical and laboratory data. Further studies on the optimal therapeutic approach are needed in order to understand the best first-line regimen, the utility of biomarkers for the definition of therapy duration and treatment of complications.

## 7. Methods

We conducted a review of recent literature by examining the MEDLINE (Medical Literature Analysis and Retrieval System Online) database using the search engines PubMed and Google Scholar. The keywords used were “osteomyelitis”, OR “bone infection”, OR “septic arthritis”, AND “p(a)ediatric” OR “children”. We included clinical trials, observational studies, reviews and meta-analyses on acute haematogenous osteomyelitis and septic arthritis in children. The exclusion criteria were patients older than 18 years, non-acute or non-haematogenous infections, case series with fewer than 20 patients, articles published before 2005, and non-English language articles.

## Figures and Tables

**Table 1:** Most common pathogens causing bone and joint infection in children and recommended first-line intravenous (IV) empiric treatment of different age groups.

Age Group	Pathogen	Empiric First-Line IV Treatment
<6 months	*Staphylococcus aureus* *Streptococcus agalactiae* (<2 months) *Escherichia coli* *Klebsiella pneumoniae*, *Candida albicans* (nosocomial infections)	First/second generation cephalosporin or anti-staphylococcal penicillin + gentamicin (if age <3 months)
6–48 months	*Kingella kingae* *Staphylococcus aureus* Group A β-haemolytic *Streptococcus pyogenes* *Streptococcus pneumoniae*	First/second-generation cephalosporin Clindamycin (if local MRSA prevalence >10%)
>5 years	*Staphylococcus aureus* *Kingella kingae* Group A β-haemolytic *Streptococcus pyogenes*	First/second-generation cephalosporin or anti-staphylococcal penicillin Clindamycin (if local MRSA prevalence >10%)

MRSA, methicillin-resistant *S. aureus*.

**Table 2:** Indications and features of imaging methods in bone and joint infection in pediatric age.

Imaging	Indications	Features
Plain radiographs	Baseline in the emergency department Excluding other conditions in the differential diagnosis	Sensitivity: <20% Specificity: 80–100% Only late signs of infections are usually detected. A normal radiograph at onset does not exclude osteomyelitis.
MRI	Confirming the diagnosis and evaluating the extension of the infection to joints and soft tissues Monitoring disease progression Surgical planning	Sensitivity: 80–100% Specificity: 70–100% Gold-standard imaging test to confirm the diagnosis Less useful in multifocal or poorly localised infections
Scintigraphy	Poorly localised or multifocal disease	Sensitivity: 53–100% Specificity: 50–100% More useful in multifocal infections Does not evaluate the extent of purulent collections
CT	When MRI is not available or is contraindicated Surgical planning	Sensitivity: <70% Specificity: <50%
Us	Evaluation and monitoring of purulent collections in joints and muscles	Sensitivity: <55% Specificity: <45%

MRI, magnetic resonance imaging; CT, computed tomography; US, ultrasonography.

**Table 3:** Indications for microbiological tests.

Test	Indications
Blood culture	In every patient at initial evaluation if BJI is clinically suspected even without fever To be repeated at fever peaks if the previous blood culture is negative
Arthrocentesis (synovial fluid culture)	Easily accessible joints: in every patient at initial evaluation if SA is clinically suspected Proximal joints: in complicated/nonresponsive cases
Bone biopsy	In complicated/nonresponsive cases if a bone abscess occurs Always when orthopaedic surgery is indicated
